# Exploring the determinants of salary valuations in women's professional soccer

**DOI:** 10.3389/fspor.2026.1720173

**Published:** 2026-03-20

**Authors:** Thadeu Gasparetto

**Affiliations:** College of Applied Human Sciences, West Virginia University, Morgantown, WV, United States

**Keywords:** association football, compensation, labor market dynamics, remuneration, soccer, sports economics, wages

## Abstract

This paper examines the determinants of salary valuations in women's professional soccer, a topic that has been largely overlooked in academic research. Using a unique dataset derived from the FIFA video game series, the study analyzes how player quality, age, skills, and international reputation are associated with salary valuations using Ordinary Least Squares regression. The findings indicate that observable measures of talent, age, technical skills, and reputation are systematically related to salary valuations, broadly aligning with patterns documented in men's professional soccer. This similarity suggests increasing convergence in how player attributes are valued across genders as women's soccer continues to professionalize. The study contributes novel evidence on salary-setting patterns in women's soccer and offers insights relevant to the sport's ongoing development, particularly with respect to visibility, commercialization, and efforts to reduce the gender pay gap.

## Introduction

Professional sports economics has extensively examined salary determinants in male-dominated leagues such as the NBA (1), NFL (2), and major European soccer leagues (3). However, a significant gap exists concerning salary determinants in women's professional soccer, despite the sport's rapid global growth (4) marked by increased participation, attendance, sponsorship, and media coverage. Most studies on women's soccer have focused predominantly on the gender pay gap (5), salary dispersion and performance (6), or match attendance (7, 8) rather than the specific factors influencing player salaries within the sport. As a result, little is known about how individual player attributes are valued within women's professional soccer markets, particularly in contexts where detailed salary data are not publicly available.

Seminal works by Rottenberg (9) and Scully (10) established foundational models linking player performance to salaries in baseball, which were subsequently adapted for soccer. In European contexts, Lucifora and Simmons (11), Frick (3, 12), and Bryson et al. (13) identified key performance metrics, such as goals, assists, defensive actions, and player characteristics, as significant salary determinants. Bryson, Rossi, and Simmons (14) found that foreign players in top European leagues often earn premiums due to perceived higher quality and competitive international talent markets, aligning with earlier findings about salary inflation following the Bosman ruling. Together, this literature highlights relatively consistent patterns in how talent and market-related attributes are rewarded in men's professional soccer.

Recent literature has expanded salary models beyond traditional performance metrics. Carrieri et al. (15) demonstrated agents’ influence in negotiating higher salaries, while research on marketability shows that players with strong social media presence command premium salaries by enhancing club brand value. Scarfe et al. (16) found that superstar salaries in Major League Soccer reflect revenue-generating capacity through stadium attendance rather than purely on-field performance. Butler and Coates (17) revealed position-specific salary effects, with defensive players earning less while versatile attacking midfielders command premiums. Berri et al. (18) showed that goalkeeper salaries often reflect basic defensive statistics rather than advanced performance metrics. These findings suggest that salary determination in professional soccer reflects a combination of productivity, skills, positional roles, and broader market visibility.

Regarding gender dynamics, Coates and Webber (5) found that determinants of win-production are consistent across men's and women's teams in MLS and NWSL, suggesting that if compensated equivalently, female players would earn more than their male counterparts on average. However, existing research on gender pay gaps (19, 20) identifies systemic disparities without comprehensively analyzing specific salary determinants in women's sports. This limitation reflects both the historical underdevelopment of women's professional leagues and the scarcity of transparent, player-level salary data.

This paper addresses this critical gap by analyzing a unique and illustrative dataset that provides algorithmic valuations of player salaries and attributes for professional female soccer players. Rather than offering definitive evidence on realized labor-market outcomes, the analysis examines how player characteristics are associated with relative salary valuations in a widely used data-generating system, drawing parallels with established findings from men's professional soccer. The objective of the study is therefore to explore whether patterns documented in the men's game also emerge in women's professional soccer when evaluated through a consistent and comprehensive proxy dataset. In doing so, the paper contributes to the sports economics literature by providing indicative evidence on salary-setting logic in a rapidly growing segment of professional sport, while acknowledging the data limitations inherent in the current market structure of women's soccer.

## Methods

### Theoretical framework

The analysis of salary determination in professional soccer can be grounded in the Mincer earnings function, a foundational model in labor economics that relates an individual's earnings to their human capital characteristics, such as education, experience, and skills (21, 22). In sports economics, this model has been adapted to examine how specific player attributes, such as age, experience, and skill levels, translate into salary differentials. The core premise is that players with higher levels of human capital, reflected in better performance metrics and skills, should command higher salaries. This framework provides a basis for evaluating how various player characteristics in women's professional soccer are associated with relative salary valuations, an area that remains underexplored compared to men's soccer.

In addition to the traditional Mincer framework, this study also draws on the theory of superstar effects (23), which posits that in certain markets, a small number of highly talented individuals can command disproportionately high salaries due to their exceptional skills and the outsized impact they have on team success and revenue generation. This is particularly relevant in sports, where players’ salaries are often influenced not only by their performance but also by their marketability, international reputation, and the value they bring to their teams beyond pure athletic performance. By integrating these theoretical perspectives, this study aims to examine whether established economic theories of salary determination are reflected in algorithmic salary-setting proxies applied to women's professional soccer. This approach allows a cautious assessment of whether similar factors that drive salaries in men's soccer are equally significant in the women's game, or if alternative factors, such as marketability, play a more prominent role.

### Data

The dataset employed in this study is a unique compilation of data on professional female soccer players, consisting of 2,291 individual observations over three seasons (2022, 2023 and 2024). The source of this dataset is the FIFA video game series, which is renowned for its detailed and regularly updated player ratings and attributes. In the absence of publicly available data on individual player salaries in women's soccer, the FIFA video game dataset offers an illustrative and internally consistent proxy for empirical research in this field. The FIFA video game series derives its data from extensive scouting reports and statistical analyses used to generate relative player valuations within the game environment.

The data analyzed in this study were obtained from publicly available player attributes within the EA Sports FIFA video game database. No proprietary data, personal information, or content was extracted from FIFA's official digital platforms or services (e.g., fifa.com, FIFA Apps, or APIs). The dataset contains only aggregated and non-identifiable information about professional players as represented in the game, and its use complies with applicable copyright and data-protection laws. Importantly, the salary variable used in this study should be interpreted as an algorithmic valuation rather than an observed contractual salary, reflecting relative differences in player valuation rather than realized labor-market outcomes.

In recent years, video game data has gained credibility in sports economics as a proxy for real-world player attributes and market dynamics, particularly when official data sources are lacking. Indeed, recent research has been using video game data as a proxy of salaries and talent, such as Yaldo and Shamir (24), Butler and Coates (17), Coates and Parshakov (25), and Gasparetto and Barajas (26). Consistent with this literature, the present study relies on FIFA-based valuations to explore indicative patterns in salary-setting logic, rather than to provide definitive evidence on actual salary determination in women's professional soccer.

### Variables

The selection of explanatory variables in this study builds upon prior empirical research on salary determinants in men's professional soccer, as examined in studies by Frick (3), Carrieri et al. (15), Scarfe et al. (16), and Berri et al. (18), among others. These works have consistently highlighted key factors such as player quality, potential, and marketability as critical in explaining salary variations. However, due to the unique context of women's professional soccer and the limitations in data availability, certain variables typically included in analyses of men's soccer, such as detailed performance metrics, were omitted. In their place, this study employs standardized proxies available within the FIFA video game data (e.g., quality, potential, skills, etc.). For instance, international reputation in this study is represented by a score generated within the game, rather than by external measures such as Wikipedia visits or Instagram followers, which have been used in previous research on men's soccer. This approach allows to adapt the model to the specific data constraints and characteristics of the women's game while maintaining comparability with existing salary-determination studies.

The dependent variable (*s*) in this analysis is the weekly salary valuation of each player, as represented in the FIFA database, which serves as the key outcome this work seeks to explain. As noted above, this variable captures relative salary valuations rather than observed contractual pay. The explanatory variables include a range of factors that capture various aspects of player characteristics and market conditions. Among these are the player's overall quality (*q*), a numerical rating that reflects their general performance level, and the potential score (*p*), which indicates the highest level of performance the player can expect to achieve in the future. Age (*a*) and an age-squared (*a^2^*) term are also included to account for the potential non-linear effects of aging on salary valuations, as experience and physical condition may impact a player's earning potential.

Additional variables include the player's preferred foot (*f*), which is categorized as either left or right, and weak foot skills (*w*), a rating that measures the player's ability to use their non-preferred foot, enhancing their versatility on the field. In the FIFA video game, preferred foot captures laterality (left- or right-footedness), while weak foot is a continuous rating that measures proficiency with the non-preferred foot. The two variables, therefore, capture distinct attributes. Skill moves (*m*), another rating, reflect the player's technical ability and creativity. International reputation (*r*) is also considered, as it represents the player's recognition and marketability on the global stage, as captured within the FIFA rating system, which can influence salary negotiations. Furthermore, nationality (*N*) is included as a categorical variable to capture potential salary variations based on a player's country of origin, considering factors such as international reputation and the competitive landscape for talent, and categorical variables for the clubs (*C*) for which the player plays, which account for differences in quality, exposure, and financial resources. Finally, the analysis includes categorical variables for playing position (*P*), allowing for the examination of salary valuations differences across various roles on the field. Table 1 presents the summary statistics of the continuous data used in this study, while Table 2 reports the distribution of players across leagues included in the sample, showing that observations are concentrated in top-tier women's professional leagues in Europe and North America.

**Table 1:** Summary statistics of continuous variables.

Variable	Observations	Mean	Standard deviation	Minimum	Maximum
Salary valuation	2,291	748.23	388.91	500	4,000
Ln (salary valuation)	2,291	6.53	0.38	6.21	8.29
Quality	2,291	73.44	6.54	52	91
Potential	2,291	78.81	5.39	56	94
Age	2,291	24.66	4.36	16	41
Weak foot	2,291	2.94	0.66	1	5
Skill moves	2,291	2.58	0.86	1	5
International reputation	2,291	1.36	0.80	1	5

**Table 2:** Frequency of players per league.

League	Frequency	Percentage	Cumulative
1st division women	28	1.25	1.25
Bundesliga women	303	13.5	14.74
Czech first division women	25	1.11	15.86
Damallsvenskan women	25	1.11	16.97
Eredivisie women	26	1.16	18.13
Feminine division 1	514	22.9	41.02
NWSL	324	14.43	55.46
Nationalliga a women	27	1.2	56.66
Primera division women	376	16.75	73.41
Scottish Premier league women	23	1.02	74.43
Serie A women	26	1.16	75.59
Women's super league	548	24.41	100
Total	2,245*	100	

* There are 46 players from different football leagues not mentioned in the game. FIFA includes them in national team squads but does not specify their leagues.

### Econometric modelling

The econometric analysis employs Ordinary Least Squares (OLS) regression to examine the association between player characteristics and weekly salary valuations in women's professional soccer. The dependent variable is the log-transformed weekly salary valuation as represented in the FIFA database. Four model specifications are estimated, each incorporating an increasingly comprehensive set of controls.

The first specification includes player characteristics, skills, reputation, age (and its quadratic term), and nationality. The second specification extends this framework by additionally controlling for playing position. The third specification further incorporates club fixed effects, capturing persistent cross-sectional differences across teams. The fourth and final specification adds season fixed effects to control for common time-specific variation in salary valuations. The final model serves as the primary reference for interpretation of the results.

These specifications allow for an assessment of how estimated associations between player attributes and salary valuations evolve as additional sources of heterogeneity are accounted for. The full specification provides the most comprehensive representation of salary valuation patterns within women's professional soccer, capturing both traditional correlates such as player quality and age, and additional attributes related to technical skills, reputation, positional roles, and team affiliation. Given the skewed distribution commonly observed in salary data, particularly in professional sports, where a small number of elite players receive disproportionately high compensation, the salary variable is log-transformed to improve interpretability and reduce the influence of extreme values.

The analysis pools observations across three seasons (2022–2024). Club fixed effects are included in Models 3 and 4 to account for persistent differences across teams, while season fixed effects are included in the final specification to control for common temporal shifts in salary valuations. To ensure robust inference, standard errors are clustered at the league level (*n* = 12), accounting for potential heteroskedasticity and within-league correlation in the error terms.

The general form of the econometric model used in this study can be expressed as follows:

$$ln(s_{it})=\beta_{0}+\beta_{1}q_{it}+\beta_{2}p_{it}+\beta_{3}a_{it}+\beta_{4}a_{it}^{2}+\beta_{5}f_{i}+\beta_{6}w_{it}+\beta_{7}m_{it}+\beta_{8}r_{it}+{N^{\prime }}_{i}\delta +{P^{\prime }}_{i}\theta +{C^{\prime }}_{i}\phi +\gamma_{t}+\epsilon_{it}$$

Where:

- $s_{it}$ denotes the weekly salary valuation of player *i* in season *t*;
- $q_{it}$ is overall player quality, $p_{it}$ is potential, $a_{it}$ is age, and $a_{it}^{2}$ is age squared;
- $f_{i}$ captures the player's preferred foot and is time-invariant;
- $w_{it}$ measures weak foot ability, $m_{it}$ denotes skill moves, and $r_{it}$ captures international reputation as rated within the FIFA system;
- $N_{i}$, $P_{i}$, and $C_{i}$ are matrices of dummy variables capturing nationality, playing position, and club affiliation, respectively;
- δ, θ, and ϕ are vectors of coefficients associated with these dummy-variable matrices;
- $\gamma_{t}$ denotes season fixed effects;
- $\beta_{0}$ is the intercept term; and
- $\varepsilon_{it}$ is the error term.

## Results

Table 3 displays the empirical results of the four OLS regression specifications described in the econometric modelling section. Models 1 to 4 progressively incorporate additional sets of controls, with Model 4 including player characteristics, nationality, playing position, club fixed effects, and season fixed effects. Model 4 represents the most comprehensive specification and serves as the primary reference for interpretation.

**Table 3:** Regression outputs.

Variables	Model 1	Model 2	Model 3	Model 4
Quality	0.0251***	0.0254***	0.0230***	0.0227***
	(0.00381)	(0.00330)	(0.00356)	(0.00355)
Potential	0.00439	0.00498	0.00240	0.00246
	(0.00391)	(0.00365)	(0.00280)	(0.00276)
Age	0.0916***	0.0918***	0.0991***	0.0994***
	(0.0241)	(0.0209)	(0.0218)	(0.0213)
Age^2^	−0.00156***	−0.00154***	−0.00167***	−0.00168***
	(0.000462)	(0.000408)	(0.000404)	(0.000396)
Preferred Foot: Right	4.64e-05	-0.0118	−0.0124	−0.0118
	(0.00770)	(0.0112)	(0.0104)	(0.0101)
Weak Foot	0.0174**	0.0151**	0.0141*	0.0142*
	(0.00580)	(0.00591)	(0.00750)	(0.00750)
Skill Moves	0.0564***	0.0343***	0.0381***	0.0387***
	(0.00230)	(0.00780)	(0.00846)	(0.00819)
International Reputation	0.154***	0.151***	0.148***	0.149***
	(0.0171)	(0.0177)	(0.0159)	(0.0159)
Nationality	Yes	Yes	Yes	Yes
Playing Position	No	Yes	Yes	Yes
Club FE	No	No	Yes	Yes
Season FE	No	No	No	Yes
Constant	2.566***	2.536***	2.846***	2.893***
	(0.413)	(0.377)	(0.380)	(0.371)
Observations	2,245	2,245	2,245	2,245
*R^2^*	0.791	0.807	0.824	0.825
Adjusted *R^2^*	0.783	0.797	0.809	0.809

Clustered standard errors at the league level in parentheses.

Notes: ***p < 0.01; **p < 0.05; *p < 0.10.

Across all specifications, overall quality exhibits a positive and statistically significant association with salary valuations. In the full model (Model 4), the coefficient on quality is 0.0227 (*p* < 0.01), indicating that a one-point increase in overall quality is associated with approximately 2.27% higher salary valuations, holding other factors constant. The magnitude of this coefficient declines relative to simpler specifications, reflecting the inclusion of additional player attributes and fixed effects.

The coefficient on potential is statistically insignificant across all four specifications, suggesting no systematic association between potential ratings and salary valuations once observable player characteristics are controlled for. Age displays a quadratic relationship with salary valuations. In the full specification, the age coefficient is positive (*β* = 0.0994, *p* < 0.01), while the age-squared term is negative (*β* = −0.00168, *p* < 0.01), indicating diminishing returns to age. The implied turning point occurs at approximately 29.6 years.

Preferred foot shows no statistically significant association with salary valuations in any specification. In contrast, weak foot ability is positively associated with salary valuations, with a coefficient of 0.0142 (*p* < 0.1) in Model 4. This implies that a player with the maximum weak foot rating earns approximately 5.68% more than a player with the minimum rating. Skill moves also display a positive and statistically significant relationship with salary valuations in the more comprehensive specifications, with a coefficient of 0.0387 (*p* < 0.01) in the full model. International reputation shows a strong and stable positive association with salary valuations across all specifications. In Model 4, the coefficient is 0.149 (*p* < 0.01), indicating a substantial salary premium associated with higher reputation scores. The inclusion of playing position, club fixed effects, and season fixed effects in Models 2 through 4 leads to incremental increases in explanatory power, with the adjusted *R*^2^ rising from 0.783 in Model 1 to 0.809 in Model 4. Finally, joint significance tests indicate that nationality, playing position, club fixed effects, and season fixed effects are each statistically significant as blocks, contributing materially to the explanatory power of the model.

## Discussion

The results demonstrate that individual player quality, skill, age, and reputation are systematically associated with salary valuations in women's professional soccer. These findings reveal substantial alignment with salary determinants documented in men's soccer, suggesting a broad convergence in how observable player attributes are valued across genders.

The persistent significance of overall quality across all specifications confirms findings from Lucifora and Simmons (11) and Frick (3) in men's European soccer, as well as recent work by Coates and Webber (5) and Berri et al. (18), all of which identify talent as a fundamental component of salary determination. The present study indicates that this relationship also characterizes women's professional soccer, with quality aggregating various position-weighted attributes that represent player talent within the FIFA rating system.

The insignificance of potential in the final model, despite being a novel inclusion in salary determination research, suggests two plausible interpretations. Either clubs do not pay premiums for uncertain future expectations, or potential is already captured through correlated attributes such as overall quality, age, and skill moves. This finding contrasts with typical labor market models where expected future productivity commands premiums, potentially indicating a more present-focused valuation approach in women's soccer as reflected in algorithmic salary-setting proxies.

The quadratic age–salary relationship, peaking at 29.6 years, closely mirrors Scarfe et al.'s (27) findings in men's soccer of 30.3 years. This similarity suggests that the experience–salary trajectory follows comparable patterns across genders, with both markets valuing accumulated experience until players reach their late twenties, after which the associated salary premium diminishes. This parallel challenges assumptions that career trajectories in women's soccer fundamentally differ from men's, at least with respect to the age–earnings profile.

The weak foot premium of 5.68% for maximum-rated players aligns conceptually with Bryson et al. (13), who documented premiums of 18.6% in European men's soccer and 13.2% in the Bundesliga for two-footedness. While the magnitude is smaller in women's soccer, the directional finding confirms that ambidexterity is valued across both versions of the sport, rewarding versatility and tactical flexibility.

The positive association between skill moves and salary valuations corroborates findings from Frick (12), who demonstrated that individual performance metrics and subjective ratings significantly influence salaries in men's soccer. This result also aligns with Zaytseva and Shaposhnikov (28), who highlighted that technical skills affecting offensive impact are crucial for salary determination, and Kempa (29), who showed that specialization in specific tasks, including technical abilities, leads to higher remuneration. Collectively, these findings suggest that technical creativity and game-changing abilities are consistently rewarded attributes in both men's and women's professional soccer.

The robust association between international reputation and salary valuations supports Rosen's (23) seminal theory that earnings among professional athletes follow a power law driven by visibility and reputation rather than solely performance. This finding parallels Carrieri et al.'s (15) evidence that popularity dominates performance in determining high salaries among male soccer players. Additionally, Yadav and Dhayanithy (31) demonstrate that reputation influences player retention and commitment, reinforcing the premium associated with global recognition. The present results indicate that marketability and brand-enhancement value are also reflected in salary-setting patterns in women's soccer.

The observed convergence in salary correlates between male and female soccer likely reflects the ongoing globalization and professionalization of women's leagues. As women's soccer gains visibility and commercial relevance, market mechanisms that have long shaped the men's game appear increasingly relevant to the women's side. This process may also be driven by the transfer of managerial practices, scouting frameworks, and player evaluation systems from men's to women's teams.

However, despite these documented similarities, the commercial and cultural context surrounding women's soccer may place different emphasis on factors not explored in this research. While patterns are similar, the magnitude of estimated associations may differ, reflecting distinct stages of league development and variation in financial investment across women's competitions. Further research exploring these nuances and investigating additional mechanisms is encouraged to deepen the understanding of salary determination in this rapidly evolving market.

### Contributions

This work represents the first exploration of salary determinants in women's professional soccer, providing novel and timely insights into the economic forces shaping this rapidly evolving industry. The findings show that talent, age, skills, and reputation are systematically associated with salary valuations, indicating that salary-setting patterns in women's soccer closely resemble those documented in men's professional soccer. This alignment with established economic theories by Mincer (21) and Rosen (23), as well as recent empirical research by Coates and Webber (5) and Berri et al. (18), highlights the relevance of these frameworks for understanding salary determination in professional sports more broadly.

The recognition and reward of players’ talent and skills in salary valuations is encouraging, suggesting that athletes can anticipate greater transparency and consistency in how their contributions are valued throughout their careers. This provides a foundation for the broader development of women's soccer, supporting salary-setting practices that evolve alongside the sport's increasing popularity and professionalization.

Notably, the strong association between reputation and salary valuations indicates that women soccer players are valued for their marketability in addition to on-field attributes. This underscores the importance of boosting attendance, audiences, and overall awareness of women's soccer, as increased visibility may translate into higher revenues and, in turn, improved earning opportunities. Such dynamics have the potential to contribute to narrowing the gender pay gap and to supporting the long-term growth and sustainability of the women's game.

### Limitations and further research

While this study provides valuable insights, several areas for future exploration remain. The use of FIFA video game data as a proxy for real-life performance and salaries is pragmatic given the lack of available real-world salary data for women's soccer. As with any proxy-based approach, these valuations should be interpreted with caution, as they may not fully capture the complexity of actual player performance or contractual salary-setting processes. Nevertheless, they offer a consistent and comprehensive dataset that enables novel empirical analysis in a context where alternative data sources are scarce. Future research could incorporate real salary data as it becomes available, thereby validating and extending the findings presented here.

An additional limitation relates to the relatively high explanatory power of the estimated models. The high adjusted R² values likely reflect the structured and algorithmic nature of salary valuations in the FIFA database, which are constructed using a limited set of observable player attributes. As such, the results should be interpreted as capturing the internal logic of an algorithmic valuation system rather than the full complexity of real-world salary-setting processes, where unobserved bargaining, institutional, and contractual factors play a larger role.

The three-season timeframe provides a focused examination of recent trends in women's professional soccer. While longer panels might capture extended dynamics and career trajectories, this period allows a timely contribution to current understanding of salary valuation patterns. Future research could explore longer horizons as additional data become available, though the findings within this window remain informative and policy-relevant. The focus on top leagues addresses the most competitive and financially significant markets in women's soccer. While results may not generalize to smaller or less-developed leagues, the insights are directly relevant to the sport's most influential segments. Further research might examine whether similar patterns emerge in lower-tier competitions or emerging markets.

From an econometric perspective, the analysis relies on standard errors clustered at the league level, reflecting the institutional structure of the data. While this approach accounts for within-league correlation, the relatively small number of leagues (*n* = 12) suggests that inference should be interpreted with appropriate caution. Future research using richer longitudinal data could explore alternative clustering strategies, such as at the player level or club–season level, to further strengthen statistical inference.

Future research could integrate real-life performance data, such as match statistics, injury history, and social media presence, to provide a more holistic view of salary determination, bridging the gap between in-game proxies and real-world factors. Additionally, variables shown to be significant in men's soccer but unavailable here, such as agent power (15), nationality effects (14), position premiums (17), and even physical attractiveness (30), could further enrich understanding of salary-setting processes. More elaborate econometric techniques, such as quantile regression (14) or non-parametric approaches (27), may also capture heterogeneity across the salary distribution. Taken together, these limitations highlight several promising avenues for future research building on the foundation established in this study**.**

## Data Availability

Publicly available datasets were analyzed in this study. This data can be found here: The data used in this study were collected from the publicly accessible SoFIFA database (https://sofifa.com), which compiles player attributes from the FIFA video-game series by Electronic Arts. No data from FIFA's official digital platforms was accessed. The cleaned dataset used for analysis will be provided as Supplementary Material upon publication.
